# Implications of Ebola virus disease on wildlife conservation in Nigeria

**DOI:** 10.11694/pamj.supp.2015.22.1.6617

**Published:** 2015-10-11

**Authors:** Adeniyi Olugbenga Egbetade, Adekayode Olanrewaju Sonibare, Clement Adebajo Meseko, Omotola Abiola Jayeola, Ebenezer Babatunde Otesile

**Affiliations:** 1 College of Veterinary Medicine, Federal University of Agriculture, Abeokuta, Nigeria; 2 Regional Centre for Avian Influenza and Transboundary Animal Diseases/ Virology Department, National Veterinary Research Institute, Vom, Nigeria; 3 College of Environmental Resources Management, Federal University of Agriculture, Abeokuta, Nigeria

**Keywords:** Ebola Virus Disease, conservation strategies, re-emerging diseases, Nigeria, wildlife

## Abstract

The recent Ebola Virus Disease outbreak in some West African countries spanning from late 2013 and currently on as of 13th March, 2015 is the most widespread and fatal with human mortality that has surpassed all previous outbreaks. The outbreak has had its toll on conservation of endangered species. This portends danger for the wild fauna of the country if proactive measures are not taken to prepare grounds for evidence- based assertions concerning the involvement of wild species. To this end, there is an urgent need for sweeping census of reserves, national parks and wetlands. As well as the creation of a system involving reportage by sectors like the industries (extractive and construction) including persons and organisations involved with wildlife related activities. This documentation of die offs and unusual events to collaborating institutions, will help in monitoring trends which hitherto would have gone unnoticed. The importance of bats and primates in agriculture and public health via consumption of vermin insects and seed dispersal cannot be over-emphasized. There is the need for caution on the tendencies to destroy indicator species which could be silent pointers to emerging or remerging health and environmental issues. Wildlife resources are still reliably useful and caution is advised in the use of blanket destructive policies like fumigation of caves, indiscriminate culling and poisoned baits to destroy supposedly Ebola Disease Virus wildlife reservoirs. This paper highlights the immediate conservation problems and likely future implications of Ebola saga in Nigeria. It tries to identify the gaps in wildlife researches and makes recommendations for probable workable conservation strategies.

## Commentary

The current Ebola scourge in West Africa is the most devastating and widespread of any outbreak either as Ebola or any infectious diseases in recent past. This is with attendant human mortalities of greater than 7000 as of Dec, 2014; 9000 as at February, 2015 [1]. The people at immediate risk in the event of EVD outbreaks are hunters, gatherers and allied workers, wildlife rangers, veterinarians, porters, field biologists and tourists. Others include workers in laboratories and research institutes, hospital care givers (for susceptible group above) and immediate families and support staff of afore mentioned group of people [2]. Wildlife conservation in West Africa has been majorly plagued by human indifference to catalogue of challenges like habitat-destruction, fragmentation and degradation, Hunting activities targeting wildlife for commercial to subsistence bush meat, pet trade and trophy hunting, pressures from urban development leading to intensive logging and incursion into wild habitats/protected areas. Currently, pathogens like Ebola virus could be responsible for direct mortality of susceptible animals in the wild and indirect population annihilation by unfavourable policies that may target reservoir animals in the wild. Re-emerging diseases in the last decades have mainly sprouted from sylvatic origins [3, 4]. The current EVD outbreak in West Africa is the most widespread and fatal with human mortality which surpassed all previous outbreaks [2, 5] The wild reservoir of the virus was unknown during the first outbreak [6]. EVD being a category “A” pathogen [7] is capable of destabilizing fragile economies and frail health care systems as obtainable in sub-saharan Africa. The impact of EVD on conservation of wild species especially the ones fingered in the maintenance of the virus is overlooked in the scheme of post outbreak plans. Confirmed cases spread across 3 continents and this will be the first spread of EVD out of Africa [4]. A mix of corruption, poverty, fear and dysfunctional amenities is perpetuating the outbreak in West Africa [8] and current outbreak is therefore a wake-up call to arrest the tide of decadence in norms and values. This also applies to attitudes toward wildlife conservation. Bats and primates, the two major suspected reservoir animals contribute immensely to ecological health and agriculture via consumption of vermin insects and seed dispersal [9], thus contributing to agricultural productivity, economy and national development. They are also indicators for ecological issues-Climate change. Some specific contribution of bats to ecology include being biological pest control, aiding forest rejuvenation via plant pollination and seed dispersal, guano mining potent organic fertilizer (NPK), ethno-medicine, aesthetics-landmarks, cultural values, bat watching/eco-tourism, research and education.

However the species also incriminated to be EVD reservoirs, do have their downsides namely being majorly pathogen reservoirs, damages to crop and building and nuisance accidents like plane strikes and bites/ injuries when handled inappropriately or impeded. The EDV RNA has been detected in implicated bat species namely Epauletted fruit bat (Epomophs franqueti), Little collared fruit bat (Myonycentris torquata) and Hammer headed bats (Hypsignathus monstrous) [4] which are found in Nigeria and the virus had been detected in all three. However, there is a dearth of information on the status of known reservoirs of EVD in Nigeria. Currently there is no ecological (population and migratory) data of known or presumed EVD reservoirs and dead end hosts. Though absence of documentation does not denote non occurrence; this however has led to blanket and often misleading statements on wildlife disease status in Nigeria. It has been estimated that a single colony of 150 big brown bats (Eptesicus fuscus) in Indiana has been estimated to eat nearly 1.3 million pest insects each year. Other estimates suggest that a single little brown bat can consume 4 to 8 g of insects each night during the active season [10]. It is also postulated that bat’ population declines in North America translates to spending extra US$ 3.7 billion/year [9]. In Nigeria, the monetary value of bats in agriculture or health has not been calculated/ documented despite their abundance in the wild and intra city. In Nigeria we ask if the outbreak of EVD was a good omen for Nigerian wildlife. We observed though not measured a reduction in incursion into protected areas, reduction in hunting pressures on wildlife. There was an obvious reduction in demand for bush meat consumption to the point that the National Association of Bush meat Sellers had to organise a public consumption of their games in public (all in an effort to boost demands). This also prompted a renewed vigour to take proper care of animals in captivity as owners do not want the tag of having animals with disease on their facilities. Could this brief reprieve trigger recovery of wild populations of if had lasted longer? We searched through literature and out of fifty one documented proofs of EVD in African wildlife, none originated from Nigeria with only Congo, Gabon, Cote d'Ivoire and Ghana contributing. In these countries bats, gorillas, chimpanzees and duikers have tested positive [4]. Absence of data from Nigeria however does not give a scientific status of EVD in Nigerian wild. These data are from countries with index cases of human outbreaks, with the exception of several sero-positive bats from a survey in southern Ghana [4]. It has been observed that mortality in the wild from EVD infection occurs during the drier months of the year. At this time, food is scarce and increased contacts and competition between and within primate colonies (pers observation AOE). There are changes in immune functions in bats around this period [9]. This is also the peak of hunting activities by humans who subsequently come in contact with infected animals Some immediate observable impacts of EVD in Nigeria on wildlife conservation include the following: there was reduction in number of visitors to wildlife parks and zoological gardens in West Africa which would have resulted into diminishing income for zoos, parks and communities; there were introduction of EDV based preventive measures and general hygiene education in Nigerian zoos, wildlife parks and sanctuaries; suspension of field researches involving primates and bats. Lack of community support for conservation outfits in their localities; mob actions, false accusation and slaughter of legally and illegally kept primates; call to fumigate the forests in some quarters; felling of trees which hitherto served as windbreaks where bats were roosting (with bats) in neighbourhoods; evolution of hitherto silent/passive associations like National Association of Hunters, Bush meat Sellers Association of Nigeria.

We postulate that some of the likely impacts of EVD may include pressure to import food into the EVD ravaged countries leading to aggravated incursion, bush clearing and farming within protected areas with wildlife. We foresee an unusual and unexpected interplay of pathogens amongst the “drivers” of disease emergence Wildlife resources are vital components of our ecology, caution is therefore advised on the use of blanket destructive policies like fumigation of caves, indiscriminate culling, use of poisoned baits to destroy supposedly wildlife reservoirs of EVD The balance in the ecosystem cannot be rocked by taking out elements of wild fauna presumed to be reservoirs of EVD. The ongoing EVD outbreak buttresses the fact that an outbreak anywhere could be a risk everywhere. Baseline data from sustained surveillance will boost our preparedness for emergencies There is an urgent need for proactive measures to generate evidence- based comments assertions concerning the involvement of wild species in Nigeria. To this end, we propose sweeping census of reserves, national parks and wetlands. We advocate for continued surveillance and periodic reviews and a regulation of hunting for bush meat. A passive surveillance system involving reportage on wildlife by sectors like mining, construction, hunting and ecotourism to designated units (zonal and regional) will suffice to fill the gaps. This will ensure documentation of die offs and unusual events which might hitherto go unreported or unnoticed to collaborating institutions. This will lead to establishment of a trend from ground zero. An adaptation of schemes like Canadian Cooperative Wildlife Health Centre (veterinary institutions, epidemiological divisions and research laboratories) may have numerous gains for epidemiological studies of diseases in wildlife. Are there benefits from harvesting sera from previously exposed non human primates? The West African outbreak underscores the need for investments by government and corporate entities into social infrastructures, development of multi disciplinary research units, holistic implementation of policies on research and infrastructural development.
